# Enhancing Health Care Delivery through Ambient Intelligence Applications

**DOI:** 10.3390/s120911435

**Published:** 2012-08-24

**Authors:** Sokratis Kartakis, Vangelis Sakkalis, Panagiotis Tourlakis, Georgios Zacharioudakis, Constantine Stephanidis

**Affiliations:** 1 Institute of Computer Science of the Foundation for Research and Technology—Hellas (FORTH), N. Plastira 100, Vassilika Vouton, GR-70013, Heraklion, Crete, Greece; E-Mails: sakkalis@ics.forth.gr (V.S.); ptourlak@ics.forth.gr (P.T.); gzaxar@ics.forth.gr (G.Z.); 2 Department of Computer Science, University of Crete, P.O. 2208, Knossos Avenue, GR-71409, Heraklion, Crete, Greece

**Keywords:** Ambient Intelligence (AmI), e-health, smart patient rooms, user interface development, smart hospital, health care

## Abstract

This paper presents the implementation of a smart environment that employs Ambient Intelligence technologies in order to augment a typical hospital room with smart features that assist both patients and medical staff. In this environment various wireless and wired sensor technologies have been integrated, allowing the patient to control the environment and interact with the hospital facilities, while a clinically oriented interface allows for vital sign monitoring. The developed applications are presented both from a patient's and a doctor's perspective, offering different services depending on the user's role. The results of the evaluation process illustrate the need for such a service, leading to important conclusions about the usefulness and crucial role of AmI in health care.

## 1. Introduction

During the past decade technology has gradually been moving towards Ambient Intelligence (AmI) environments aiming to help inhabitants in everyday life [1–3]. AmI supports the pervasive diffusion of intelligence in the surrounding environment, through various wireless technologies (Zigbee, Bluetooth, RF, WiFi) and intelligent sensors. These environments integrate various hardware and software technologies, allowing users to control electrical and electronic devices automatically or manually. To simulate the needs of such an environment, a development laboratory (sandbox) has been equipped with various wireless and wired sensor technologies [4] specifically tailored for home automation. The case study researched to form an understanding of the significance of: (a) the accuracy, (b) the data loss and rate, (c) the radio range, penetration and attenuation, (d) the harmful radiation level, as well as (e) the total cost of hardware infrastructure [4]. The first application developed by the authors in the context of AmI was CAMILE [5], which allows users with disabilities to control lights easily through an accessible user interface. Subsequently, a new system was created that generates accessible Graphical User Interfaces (GUIs) automatically allowing user to control every smart appliance [6].

This paper extends the idea of environment control applied to clinical practice, by presenting a Smart Patient Room that allows users to control the environment and efficiently use the hospital facilities, while at the same time helping nurses and doctors by automating their clinical routines. There are various devices that a patient can control using an application tailored to a touch pad device, including lights, the set of blinds, TV sets and the bed. Such a hospital room is simulated in our laboratory sandbox located in FORTH-ICS premises. The proposed environment can be easily adapted to support disabled patients in a home environment, as well.

This paper is structured as follows: Section 2 discusses related work. Section 3 presents an example scenario illustrating the use of the developed smart hospital room. Section 4 describes the hardware infrastructure. Section 5 describes the software architecture and presents the Graphical User Interface (GUI) of the application. Section 6 reports the results of the evaluation process, which involved seventeen participants of different computer expertise and ages. Section 7 concludes the paper and presents future work directions.

## 2. Related Work

Improving the quality of health care and ensuring substantial cost savings is the target of most of e-health applications [7,8]. Remote patient monitoring (vital sign monitoring [9,10], softcopy radiological film review [11,12], *etc.*), condition specific diagnostics and treatment [13] were the first applications appearing in the domain, mainly addressing the need to support diverse clinical requirements. Also, in the domotics field much work has been conduct to provide assistive environments, e.g., smart homes or hospitals, using RFID, monitoring cameras and environmental sensors [14–16]. Through the above efforts, it became apparent the need to provide integrated services able to interconnect all the fragmented available e-health systems and automation systems and sensors by a binding architecture [17,18].

Wireless technologies and smart environments play a fundamental role in helping this integration vision by providing natural and user-friendly ways of coping with the surrounding environment. Going back to the work conducted by Eggen *et al.* in 2003 related to home environments [19], a crucial finding was that people, apart from the desire to be surrounded by pleasant atmosphere and decorations, also stressed the need of feeling in control, thus contrasting the quest for autonomous behavior typical of many AmI research efforts. On the other hand, nurses want to attend their patients and not the technology, so the smart environment should ease their workload and not bring additional administrative tasks [20]. In this direction, this paper proposes a smart room adaptation in a clinical setting that facilitates both a disabled patient to act independently and fully control the patient room with no further support, as well as the nurse's and the doctor's monitoring routine, by automatically providing an accurate patient profile and medical measurement logging. Such technologies and approaches further promote clinical quality of care, while sustaining at the same time patient's independence and quality of life, especially to those with chronic diseases who must travel regularly for minor typical examinations [21]. Furthermore, by enabling the “virtual visit” of a medical doctor, this work has the potential to reduce costs for the healthcare system, unnecessary patient travelling, and social costs for the families and relatives of the patient.

Past studies have shown that patients are willing to pay for such improvements to healthcare [22]. In this context, understanding what users (patients and doctors) are expecting from future hospital rooms is an increasing driver of human-home interaction research, with the major aim to move from technology-driven to user-driver approaches. Thus, a key aspect of this work is that it is user-centered, relying on end-user evaluation for improving the initial design. Another key point of this work is the fact that, being implemented as a service oriented architecture (SOA), it not only allows easy extension for future needs, but also provides the ability to adapt the environment for specific cases and provide different interfaces for patients with disabilities, visual or hearing impairment *etc.*

## 3. Use Case Scenario

### 3.1. Design Criteria

Most of the mobile systems available on the market today are focused mostly on clinical documentation, computerized physician order entries, EMR management, real-time data gathering and transmission of critical patient information at the point of care. To our knowledge, there is no system available capable of providing both patient and physician services at the same time by combining smart control of the patient's environment with specialized clinical services. Both are found to be of paramount importance in improving the overall quality of patient care. Furthermore, during the last few years with both consumer and business-focused tablet devices hitting the market, it's essential for healthcare providers to pay attention to integrated features and ergonomics. The former concerns were our driving force while implementing the system.

The presented smart environment is designed to cover as many different needs as may emerge in a healthcare environment, such as a hospital room, a primary care clinic or even in a patient's house. To this end, adaptive technologies and requirements must fit together to better serve diseased or disabled people that have different—and many times conflicting—needs. At the same time, the healthcare environment should be able to fulfill also the requirements of the medical personnel and be easily translated to existing clinical setting. Eventually it becomes obvious that even a setting which is totally personalized for one patient may prove inadequate to serve another individual who shares the same health problem. In order to compromise the different requirements, we decided in the design process not to base the implementation on a specific patient category or health problem, even if some of the technologies are targeted mainly on specific health domains, but aim to provide a cost-effective, widely applicable and efficient solution that may cover many different patient categories and leave the tailoring of the system as an installation decision when a similar system is deployed in a real clinical setting.

Another important consideration was the extensibility of the system, in order to be able to incorporate results of our ongoing research. Technology nowadays has matured enough to support control and interaction of a person with his environment by using gestures, eye tracking, voice recognition and brain signals. Also, the user interface design can be adapted to serve people with vision problems or movement disabilities. So, the automation and integration of many functionalities, was done not only based on the ground of necessity based on present needs, but also to support emerging AmI functionality of the future.

Additionally, the integration of the offered services was not done solely for the sake of automation or to enhance the user experience, but also for tracking, contextualization and smart fusion of different sensors. For example, the automated acquisition of medical measurements, such as the blood pressure, heart rate, body weight change, temperature *etc.* is not performed only in order to assist the nursing staff in their daily routine, but also to be able to transparently integrate the measurements with the electronic health record (EHR) of the patient, to compare the measurements with thresholds set by the doctors and to detect alerting changes or patterns [10]. The driving factor behind this research was the fact that the added value of an integrated environment is much higher than the sum of its constituents. And this is also the reason why we based the implementation on an integrating middleware that is able to connect different services, than simply hard-wiring specific functionalities and technologies focusing on a pre-defined category of patients, thus limiting the future potential.

In summary, the proposed system focuses on:

- Modular Design (may be easily generalized to cover different healthcare needs and domains/clinics) with no need of changing the clinician workflow.
- Easy and cost-effective translation to the clinical setting. Existing appliances may be adapted to fit in the solution. No manufacturer specific hardware is presumed.
- Efficient utilization from both the patient and the clinician (dual GUI is provided and dynamically adapted to the different needs of the clinician (doctor's UI view) and patient (patient UI). Hence, there is no need for the clinician to move the tablet around, thus minimizing the disinfection process, since the tablet is not moving from one patient room to another the risk to pass along infections is minimized, as well as damaging the device due to an accidental drop in the rather fluid environment of the hospital.
- Providing a User Friendly interface for elderly, disabled and diseased people.
- Allowing faster turnaround times by avoiding fragmented clinical workflows.
- Providing improved accuracy through point-of-care electronic access to the patient data allowing faster decision making.
- Allowing clinicians to access the critical information they need when they are face to face with a patient.

### 3.2. Exemplar Scenario

**Figure 1.**
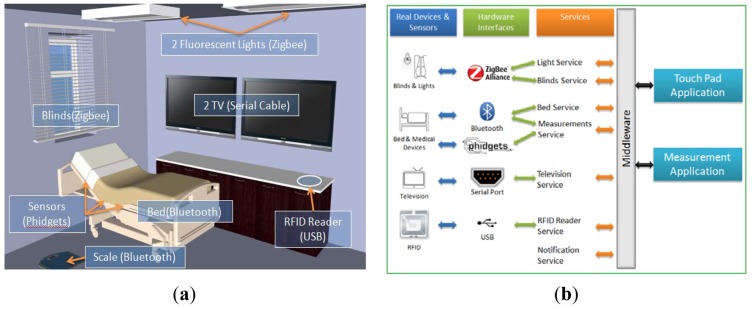
(**a**) Hardware infrastructure in smart patient room. (**b**) System architecture.

An example scenario involving a room furnished with a bed, two TV sets and a wooden storage cabinet, as depicted in Figure 1(a), illustrates our approach. In the current scenario three users participate: a patient, a doctor and a nurse.

The patient is considered as not being able to get up from bed, thus an application for a touch pad was developed to help the patient control the environment and use the available hospital facilities. The room equipment that the patient can control directly includes the lights, the blinds and the rear TV. Apart from using the available devices, the patient also has the ability to call the nurse in case of an emergency or any other specific reason, *i.e.*, changing the linen, renewing the IV bag or the urinal vessel and tidying up the room. The application also provides a scheduling mechanism that assists the patient in organizing important activities and needs, providing visual and sound alerts such as “take pills”, “doctor check-up visit” and “end of visiting hours”.

In the case of the doctor, the application aims to facilitate the visits to the patient, as well as the analysis and representation of the patient's medical data. For example, suppose that the patient is watching TV at the time the doctor comes in the room for the scheduled check-up visit. The doctor has an RFID card that can be placed on top of the wooden cabinet, resulting in two actions. First, the TV close to the patient is automatically turned on and tuned to the channel that was previously selected, while the rear TV displays relevant medical data that can be used by the doctor to collect patient information such as blood pressure, heart rate, blood-oxygen saturation (SpO2) level and standard 12-lead Electrocardiogram (ECG). The same application that is displayed in the rear TV also becomes visible on the touch pad, with the only difference that the heart graph is not displayed on the portable device. The measurements provided by the application are obtained continuously in real time, while a history of the latest relevant measurements is provided in the form of a patient log.

## 4. Hardware Infrastructure

The hardware infrastructure integrated in the Smart Patient Room is illustrated in Figure 1(a). The devices comprising the equipment used in the scenario are:

### 

#### PC

A computer installed inside the wooden furniture near the bed. All the available devices and sensors are connected to this computer.

#### TV Sets

Two 42′ LCD televisions connected to the PC via serial cables. In order to control these TVs a special serial communication protocol was implemented.

#### Controllable Fluorescent Lights and Blinds

There are two fluorescent lights on the ceiling and a window with blinds that are controlled by default through custom hardware controllers. These devices are away from the PC, thus their control mechanisms were altered in order to support data exchange with the PC through Zigbee interfaces on available USB ports.

#### Medical Devices and Sensors

In our reference implementation the supported measurements are: Blood Pressure [BP; A&D UA-767PBT Blood Pressure Monitor acquiring BP (systolic, diastolic and mean arterial)] measurements and Heart Rate (HR; Nonin Avant 4000 Digital Pulse Oximeter providing real time measurements of HR and SpO2, transmitting via Bluetooth), Blood-Oxygen Saturation (SpO2), body weight (A&D UC-321PBT Weight scale measuring the person's weight, transmitting via Bluetooth) and 12-lead ECG monitoring (Welch Allyn Cardio Perfect 12 lead ECG Recorder transmitting the recorded ECG via fiber optic cable). The patient's electrocardiogram is taken by a 12-lead ECG monitor device and is sent to a PC, where it can be visualized as a real-time electrocardiogram graph or archived as an SCP-ECG file which can be attached to the patient's Electronic Health Record (EHR).

#### Controllable Hospital Bed

A mechanical bed that allows changing the position at the height of the patient's back and feet, as well as its height and rotation angle. By default it is controlled through a panel of buttons that was replaced by a custom controller in order to communicate with the PC via Bluetooth.

#### Pressure Sensors

In order to detect when the patient lies down in bed, a set of pressure sensors were installed under the mattress and connected to the PC via USB through a Phidgets Controller Interface.

#### RFID Reader

An RFID reader was installed inside and close to the top surface of the wooden furniture near the bed. This allows the doctor to be recognized by the system in order to change the context of the display devices.

#### Touch Pad PC

A portable touch pad that hosts the application that the patient can use to control the room.

## 5. Applications and Services

### 5.1. Architecture

As illustrated in Figure 1(b), the main parts comprising the system's architecture are: a middleware, a set of services, the various devices and sensors and specialized applications. The middleware [23] is basically an AmI environment's layer that is responsible for the communication between the available devices/sensors and the applications. In order to use the devices/sensors, special low-level applications must be created that can retrieve and/or send back information to the devices/sensors. These applications are called services and the middleware takes care of delivering a service's messages and commands to other applications. A service was implemented for each of the equipped devices used in this scenario (the lights, blinds, TV, bed and RFID reader), as well as one for the measurements and one for notifications. These services make use of the aforementioned technologies (Zibgee, Bluetooth and Phidgets) and handle the respective data. The advantages of the proposed approach, as opposed to commercial solutions are: (a) the lower cost, (b) the easy and rapid integration with virtually any type of sensor, (c) the wide variety of supported wireless and wired sensors' technology, and (d) the platform independence.

Finally, two new applications were designed and developed running on top of the middleware services:

- the *Touch Pad Application*, which allows the user to control the environment, and
- the *Measurement Application*, which enables the doctor to monitor the patient's status in real time.

### 5.2. Patient's Touch Pad Application

**Figure 2.**
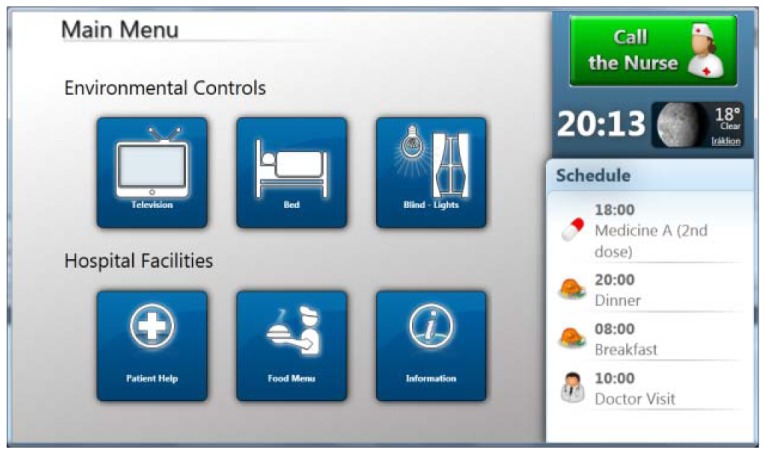
Touch pad application main screen.

The main screen of this application's user interface is illustrated in Figure 2. On the right part of this screen there are three components that are always visible, regardless of which menu is selected. The first is a big green button that the patient can use to call the nurse at any time. Below the “nurse button”, a widget presents the current time and temperature. The bottom right part of the screen is occupied by the patient's schedule that displays upcoming events sorted chronologically. For example, in Figure 2, the next event is the doctor's visit. In order to make it easier for the patient to understand the next event, an icon is associated to event types.

The left side of the application's interface is occupied by the main menu that contains two action sets, each consisting of three buttons. The first set refers to environmental controls, *i.e.*, the TV, bed and blind/lights, while the second one provides the hospital facilities, *i.e.*, patient help, food menu and information.

#### *TV Menu* [Figure 3(a)]

This menu allows the patient to turn on/off the TV, alter or mute the TV's volume, as well as change the current channel sequentially (next/previous) or individually by using a grid of buttons that contains the logo and the name of each channel. Apart from that, an extra label is presented on top of the menu to indicate the current TV state (on/off and the current channel).

#### *Bed Menu* [Figure 3(b)]

A significant problem that may occur when a patient is alone in the room is the inability to easily change the position of the bed's back. The functionality provided by this menu allows users to move up and down the bed's back and the portion at the legs' height, as well as the elevation and rotation of the whole bed. This is rather useful for doctors and nurses as well, since there is no need to lift the patient in order to alter the bed's state.

#### Blind and Lights Menu [Figure 3(c)]

As previously described, there are two fluorescent lights and a set of blinds in the room. A set of buttons allows the patient to open/ close the blinds at any time regardless of their current movement or position. Another set of buttons provides the ability to change the intensity of the lights between 0%, 30%, 60% and 100%. These discrete values have been selected instead of allowing the user to analogically change the light intensity, mainly because intermediate values are difficult to distinguish for the human eye.

#### Patient Help Menu [Figure 4(a)]

This menu allows patients to call a nurse for a specific reason. The predefined reasons are changing the linen, the IV bag or the urinal vessel, as well as cleaning the room.

#### *Diet Menu Plan* [Figure 4(b)]

The patient's diet should be balanced and proportional to the existing medical problem as well as the patient's preferences. Thus, the patient's food menu is automatically updated on a daily basis and presents two proposed lists of different meals, lunch and dinner respectively.

### 5.3. Measurement Application

**Figure 5.**
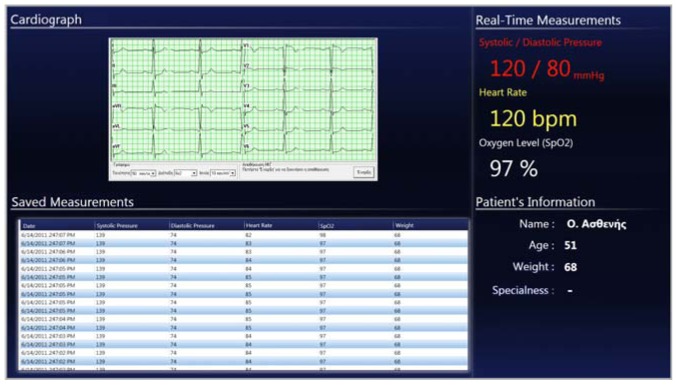
Measurement application for TV illustrating vital signs monitoring and 12-lead ECG.

During a check-up visit, the doctor can use a personal RFID card to activate the room automation system, which is adapted to his/her profile. The patient can keep watching TV in one display while the doctor uses the other in combination with the touch pad to monitor the bio-sensors' values. In Figure 5 the user interface of the measurement application that appears on the TV and touch pad is presented. A significant part of this application is the ECG component that displays data received from the 12-lead cardiograph monitor device. An extra feature of the ECG component is that it can enable cardiograph recording, so that retrieved data can be used from a different application. The other parts of the application are real-time measurements, logged measurements and patient's information. Real-time measurements consist of information on diastolic and systolic pressure, heart rate and SpO2 percentage. Each measurement is represented in a different color to help the doctor distinguish them at a glance. Logged measurements are refreshed each time a bio-sensor sends a new measurement and are stored in a list view. By clicking the header of a list view column the data is being sorted by the specific measurement value (one click for ascending and two for descending order). The provided patient information consists of name, age, and weight that is updated dynamically from the electronic scale. Patient history (e.g., previous diagnosis) and specific information (e.g., allergies) are also provided.

## 6. Evaluation

The main driving force in this endeavor was to move from approaches based on “pushing needs” to a fairer approach of “supporting needs” [24] that will likely boost the adoption of smart and flexible environments and possibly sustain increased commercial adoption of these systems. Hence, a preliminary evaluation was performed targeted to assess Smart Patient Room tablet application against the four usability principles as outlined by Booth [25]: (a) usefulness, (b) effectiveness, (c) learnability, (d) likeability or satisfaction. For that purpose, the evaluation included both a quantitative and a qualitative method of measurement. Specifically, for the quantitative analysis, the Brook's System Usability Scale (SUS) was used [26], a simple, ten-item attitude Likert scale, covering a variety of aspects of system usability, such as the need for support, training, and complexity. Apart from this, the Success Rate [27] usability metric was used to measure users' ability to complete the tasks. For the qualitative analysis the think-aloud method was used, asking the users during and after each task to comment on their experience.

### 6.1. Set-Up

All the evaluation sessions took place in the patient room. Two cameras were used to record the experiments, one for capturing the touch pad area, including system visual and speech output, as well as the user's hands (when appropriate) and one for capturing the face, body and speech of the participant. Along with each participant there were two evaluators in the room. One of the evaluators was first introducing the participant to the goals and functions of the system and then reading aloud a number of tasks that the participant should try to accomplish, while the other was mainly operating the two cameras. Only one of the evaluators would answer any raised questions and would help the participants if they got confused or stuck with a task.

### 6.2. Participants

This version of the application was targeted to adult users with no specific functional limitation. Seventeen volunteers participated in the evaluation, nine females and eight males. Each participant filled in a short questionnaire containing demographic and computer experience information. Three participants were user experts and user interfaces designers of the HCI laboratory. The age of the participants ranged from 21 to 55.

Eleven participants were regular users that use computers for emails, internet and word processing. One of them was sixteen years old. Nine of them were in the 18–40 age-group, while the last was between 41 to 55 years old.

Three participants had almost no computer experience. Two participants ranged from 41 to 55 and the last was between 21 to 40 years old.

### 6.3. Process

The process started by introducing the participants to the functions and goals of the room. After that, the objectives of evaluation were explained to them, and the process of the test was described. Each participant was then given a consent form, requesting their agreement to videotaping the session. Then, the participant's profile (e.g., age, sex, computer usage frequency) was captured by a means of a short questionnaire that was filled in through a brief interview session before the experiment started. After that, all participants were asked to perform a series of tasks as presented in Table 1.

### 6.4. Results

#### Quantitative Results

**Figure 6.**
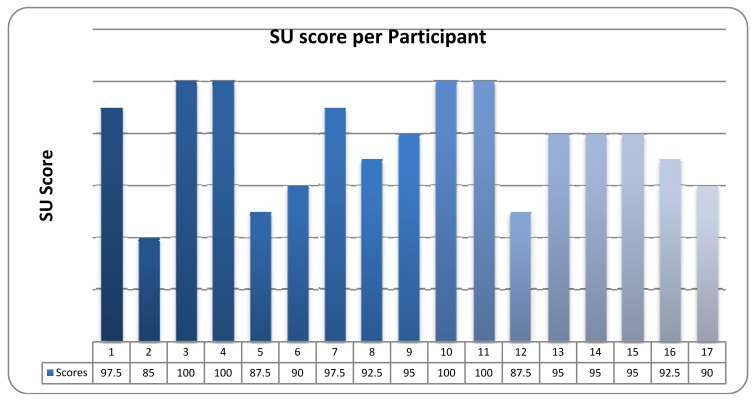
SUS Results.

The mean System Usability (S.U.) as perceived by all the participants was 94.1, which is considered as quite high. The individual calculated Total System Usability scores per participant are presented in Figure 6.

The second usability metrics was the Success Rate. In order to calculate the Success Rate, all videos were reviewed, marking each task for each participant as (a) Succeeded, (b) Partially succeeded or (c) Failed. Each score for every task was calculated by the formula:

(1) $$\frac{\begin{array}{c} \text{Participants' Success}\\ \text{for Task X} \end{array}+\frac{1}{2}\left[\begin{array}{c} \text{Participants' Partial}\\ \text{Success for Task X} \end{array}\right]}{\text{Number of Participants}}100$$

The total Success Rate, *i.e.*, the average of the scores for all tasks, was 94.1. The total score, the score and the description of each task are presented in Table 1. This score is equal to the SUS score. This produces two different conclusions: (a) the evaluation process was very successful and (b) the participants answered to the SUS questioner accurately and according to their difficulties as captured in the video.

Apart from using the SUS questionnaire and Success Rate for collecting quantitative data, the think-aloud method was also employed in order to collect rich qualitative feedback, by encouraging the participants to comment on their experience of using application.

##### Usefulness

Overall, all participants found the application very useful. The users liked the simplicity of the interface and particularly the fact that they could control all of its features with a touch of an icon. According to the users, this system would allow patients to easily control their environment and benefit from its facilities without having to get off the bed or the need for an assisting person. Most users stated that they would absolutely use that system if it was installed in a hospital. Seven users also proposed some extensions to the system, such as an integrated web browser, a media center, an e-book viewer, even games.

##### Effectiveness

The users found the touch pad very easy to use. Although placing it on an adjustable mechanical arm might have been more comfortable for the users, the purpose of the testing process was to evaluate the application and not the hardware infrastructure. The available features were well-integrated within the system, while the selected colors, icons and labels made the interface rather helpful in accomplishing the evaluation tasks. An issue that many users encountered was the lack of visual and audio feedback in some cases, such as calling the nurse for help. While fifteen users pressed the right button, they could not know if the nurse was actually informed. All users were able to perceive the state of the appliances in the environment, but most of them would prefer for the system to be updated to display the environment state too. For example, in the menu for the blinds, the users would like to see if the blinds are already open or closed by highlighting the respective menu option.

##### Learnability

The participants were not given a tutorial on how to use the system, but were instructed to use it based on intuition. After the first two tasks none of the participants had any problems navigating through the system's interface. Seventeen participants said that the application is very easy for regular users to learn, but also stated that an old person with no experience in computers would probably encounter some difficulties.

##### Likability

Sixteen users responded positively to the question “Do you think you would be using this system often if you had it in your house?” All users agreed that the user interface was visually pleasant and although it was very simple they enjoyed the fact that it provided rich functionality. Ten users also stated that they would like to have such a system in order to control other rooms of their houses too.

### 6.5. Safety and Reliability Concerns

Apart from the usability issues, a major consideration in the design of our system was the safety for the participants and the reliability in the functioning of the system, since a sensor system in the Prognostics and Health Management (PHM) domain could result in adverse events.

It is important to note that from the devices listed in Section 4, not all of them are Medical Devices under the definition of FDA [28] or the EC [29] directives. The devices which are used to acquire medical measurements, *i.e.*, the Nonin pulse oximeter, the A&D blood pressure monitor, the A&D weight scale and the Welch Allyn ECG recorder are FDA approved commercial medical devices. Also, the mechanical hospital bed is an EC approved bed. All the additions, alterations or sensor integrations which have been applied were solely for the purposes of our research and have not been validated or approved yet by any official certification body or clinical study. As a consequence, the smart environment which is described in this work cannot be used “as is” in a clinical setting without the required clinical validation for the parts of it that it might prove necessary.

As noted in the work of Cheng *et al.* [30] one of the recommended strategies, which we also employed, is the redundancy or sensors for the monitoring of the same functionality. This strategy is usual for all distributed or unreliable subsystems in order to increase the reliability and fault tolerance of the integrated system. For example, for the detection of the patient on the hospital bed we distributed several sensors in various locations under the mattress since the cost is not prohibitive for using multiple sensors for the same purpose. Also, the sensors tend to provide indications with a minor inaccuracy in their sensitivity after a period of time and they require a re-calibration, so none of the indications is considered as correct by default and the decision whether the patient is actually located on the bed or not, is taken based on a Byzantine agreement algorithm, depending on the indications of the majority of the sensors. The measurements of the medical devices are considered to be correct, since the medical devices include correction algorithms which provide error indications in case of unreliable or unstable measurements, but the wireless or wired communication with the devices is treated as unreliable and we employ a continuous polling-and-reconnect mechanism. All battery-operated devices provide indications and alerts when they run out of battery, and all devices or sensors which are connected with a power supply have been installed by trained personnel for all the necessary safety precautions. We do not use drugs or life-critical appliances in our laboratory setting.

## 7. Conclusions

The smart hospital room scenario was created as a first rapid prototype to record the needs of the patients, doctors and nurses and is part of our endeavor to take the current clinical practice and to overlay a vision of the future, to see how the addition of new technologies can have positive impact on today's healthcare. Another main challenge was to keep the cost and time low, so as to cost-effectively facilitate the conversion of real hospital room into smart rooms. The results of the recording was the creation of a new smart room not only for a hospital, but also as a room for a smart home that assists people with various diseases in their everyday life. The experience gained from the development and evaluation of the smart hospital room has confirmed that the proposed technical solution can easily transform a typical clinical ward to a smarter one, thus improving the quality of care and life, and helping patients, doctors and nurses. The conversion from an existing hospital room to a smart one is possible in an almost wire-free way, at the same time being financially affordable even for small hospitals. Additionally, the same solution can be adopted for home monitoring as well, thus widening potential market applications. Undoubtedly, future research should include more studies with a longer study period to measure the long-term clinical effectiveness of such a system by comparing our system with the standard of care.

Currently, a new version of the patient's room is under development. Patients and doctors will be able to communicate with relatives and other doctors, respectively, through video conference over VOIP by using the touch pad application and camera. Patients' data can be sent to another hospital or specialized clinic, enabling clinicians' collaboration. A smart conference room has been created that allows doctors to gather around a large-scale interactive office table in order to present, exchange and analyze relevant data. A new application for smart mobile phones, which is under development, will inform nurses about a patient's requirements, as well as send alert notifications to the doctor in case of emergency. In order to explain to a patient his/her health problem, augmented reality will be used to show in real-time over the patient's body the situation and damage at the point of interest (e.g., a broken leg).

## Figures and Tables

**Figure 3.**
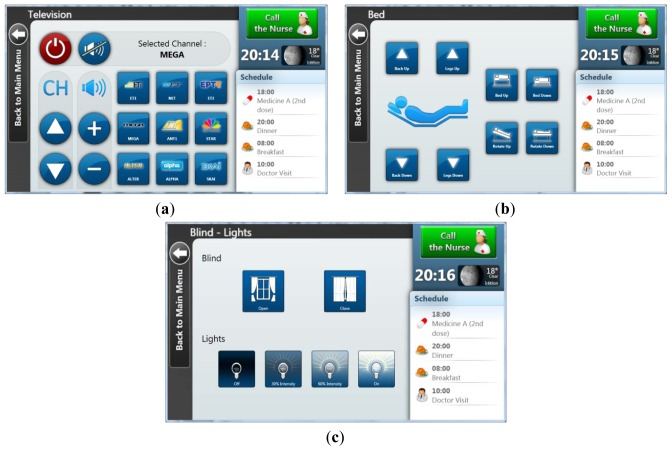
(**a**) Television Menu. (**b**) Bed Menu. (**c**) Blinds and Lights Menu.

**Figure 4.**
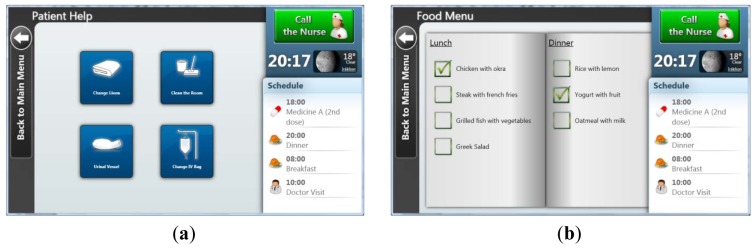
(**a**) Patient Help Menu. (**b**) Food Menu.

**Table 1.** Success rate results.

**Tasks**	**Description**	**Success Rate**
1	Turn the lights on and adjust your favorite intensity	100
2	Open the blinds	100
3	Move up the bed's back and change the position of the other parts of the bed in order to feel nice	97.2
4	Turn on TV, change the channel and turn off the volume	100
5	While watching TV, you feel sick. Call the nurse	79.4
6	What time is the dinner according to the system	100
7	Which are the available choice for the two meals and select your favorite	85.3
8	While you eat, water spilled on your sheet. Ask through the system to change the sheets	91.2
**Total success rate**		94.1
